# Editorial Note: Low-carbon governance, fiscal decentralization, and enterprise green development: Evidence from China

**DOI:** 10.1371/journal.pone.0357637

**Published:** 2026-09-08

**Authors:** The PLOS One Editors

The *PLOS One* Editors issue this Editorial Note to inform readers of the following issues that were noted after this article’s [1] publication:

- Reference 30 was retracted after [1] was submitted and before [1] was published.
- Reference 33 was retracted after [1] was published.

PLOS considers that these references are not crucial in supporting the research or conclusions reported in [1].
